# Prognostic Role of Tumor Budding in Breast Cancer Patients Receiving Neo-Adjuvant Therapy

**DOI:** 10.3390/jcm10040827

**Published:** 2021-02-18

**Authors:** Paul Mozarowski, Bhubendra Rasaiah, Melissa Reed, Alexis Lewis, Natalie Walde, Ioannis A. Voutsadakis

**Affiliations:** 1 Department of Pathology, Sault Area Hospital, Sault Ste. Marie, ON P6B 0A8, Canada; mozarowskip@sah.on.ca (P.M.); rasaiahb@sah.on.ca (B.R.); 2 Clinical Trials Unit, Sault Area Hospital, Sault Ste. Marie, ON P6B 0A8, Canada; reedm@sah.on.ca (M.R.); lewisal@sah.on.ca (A.L.); walden@sah.on.ca (N.W.); 3 Faculty of Medicine, University of Ottawa School of Medicine, Ottawa, ON KK1H 8M5, Canada; 4 Department of Biology, Algoma University, Sault Ste. Marie, ON P6A 2G4, Canada; 5 Algoma District Cancer Program, Sault Area Hospital, Sault Ste. Marie, ON P6B 0A8, Canada; 6 Section of Internal Medicine, Division of Clinical Sciences, Northern Ontario School of Medicine, Sudbury, ON P3E 2C6, Canada

**Keywords:** tumor budding, neo-adjuvant treatment, breast cancer, prognosis, marker

## Abstract

Background: Isolated tumor cells or small clusters of tumor cells observed in the vicinity of the main tumor mass in pathology sections, termed tumor budding, are common in cancers and have been associated with prognosis in some settings. This study examined the clinical associations and treatment efficacy implications of tumor budding in breast cancer patients receiving neo-adjuvant therapy. Methods: Breast cancer patients that received neo-adjuvant therapy before definitive surgical treatment in a single cancer center over a 7-year period were included, and their records were reviewed. Data extracted included patient demographics, tumor characteristics and pathologic response to treatment at surgery. The initial breast cancer biopsy before any therapy was reviewed by two pathologists, and a hot spot area was evaluated for tumor budding (defined as 1 to 5 cancer cells observed detached from the main tumor mass). Results: Seventy-five patients who received neo-adjuvant therapy (73 received chemotherapy and 2 received hormonal therapy) were included. Tumor budding was observed in two-thirds of the patients. There were no significant differences in patient (age and menopause status) and tumor (stage, histology and molecular sub-type equivalent) characteristics between the group that had tumor budding and the group that did not have tumor budding in the pre-treatment biopsy. Likewise, no statistically significant differences were observed in the frequency of complete or partial responses between the two groups. Conclusion: In this cohort of breast cancer patients receiving neo-adjuvant therapy, tumor budding was frequent, but it was not associated with tumor characteristics or pathologic responses to treatment. The value of tumor budding as a prognostic factor in the neo-adjuvant setting within the general breast cancer population could not be confirmed, but such a value in specific sub-groups deserves further investigation, given the pathophysiologic rationale and data from other settings.

## 1. Introduction

Breast cancer is a highly prevalent malignancy in women and a cause of significant morbidity and mortality [1]. It is the most prevalent cancer in women in the United States and is second after lung cancer in mortality. About two-fifths of patients are younger than 60 years old at diagnosis [2]. Progress in the detection and treatment of breast cancer in the last few decades has led to reduced mortality. However, further improvements in outcomes based on biomarkers and targeted therapies are needed, especially in aggressive sub-types, such as triple negative cancers [1,3,4].

Neo-adjuvant chemotherapy is increasingly used in locally advanced breast cancer with the aim of breast conservation and in cases with positive lymph nodes. In addition, neo-adjuvant therapy provides invaluable information regarding the chemosensitivity of cancers that may be incorporated into post-treatment algorithms. The molecular sub-type of breast cancer is a predictor of response to neo-adjuvant chemotherapy, with cancers of triple negative and HER2-positive sub-types more commonly obtaining complete pathologic responses than ER-positive cancers. However, beyond tumor sub-type, no other clinical or pathologic markers have been consistently proven to predict therapy response in the neo-adjuvant setting [5,6]. Thus, there is a need for identification and validation of such markers to guide therapeutic decisions.

Tumor budding has been observed in various cancers. The exact definition of tumor budding varies among studies, but it generally refers to a small number of cancer cells, usually up to five cells, which are detached from the main bulk tumor mass and are observed as isolated cells or small clusters of cells in histologic sections [7]. When tumor buds are located at the margins of a tumor mass, they are called peritumoral buds, and when they are located inside a tumor mass, they are called intratumoral buds [8]. Tumor budding can be identified in plain eosin and hematoxylin sections, although some investigators use immunohistochemistry for epithelial markers to increase the accuracy of budding identification. Tumor budding has been described in gastrointestinal cancers. Colorectal cancer is the gastrointestinal malignancy where tumor budding has most commonly been used as a prognostic factor [9]. In colorectal cancer, a prognostic role of tumor budding in resected stage II cancers has been confirmed. Stage II colorectal cancer patients with tumor budding had inferior overall survival compared with patients with no tumor budding [10]. In rectal cancer, tumor budding in biopsies before neo-adjuvant chemo-radiation was predictive of poor response to neo-adjuvant treatment [11]. Other cancers where budding has been studied include lung, head and neck carcinomas, as well as breast cancers [12]. In breast cancer, tumor budding has been described as a predictive marker in localized disease [13,14,15,16]. However, tumor budding has not been examined in the neo-adjuvant breast cancer setting.

This study describes the association of tumor budding with clinicopathologic factors in breast cancer patients that received neo-adjuvant chemotherapy and explores its potential value as a predictor of response.

## 2. Methods

Breast cancer patients who received neo-adjuvant therapy over a 7-year period were identified in the breast cancer section of the electronic patient database at our cancer center, and their records were retrieved and reviewed. Patient demographics and tumor characteristics were recorded in a database constructed for the study.

**Figure 1:**
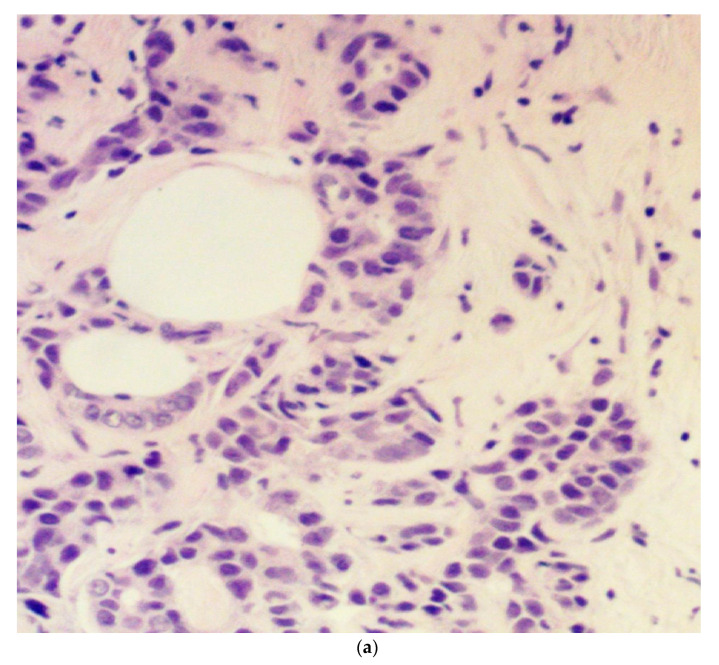

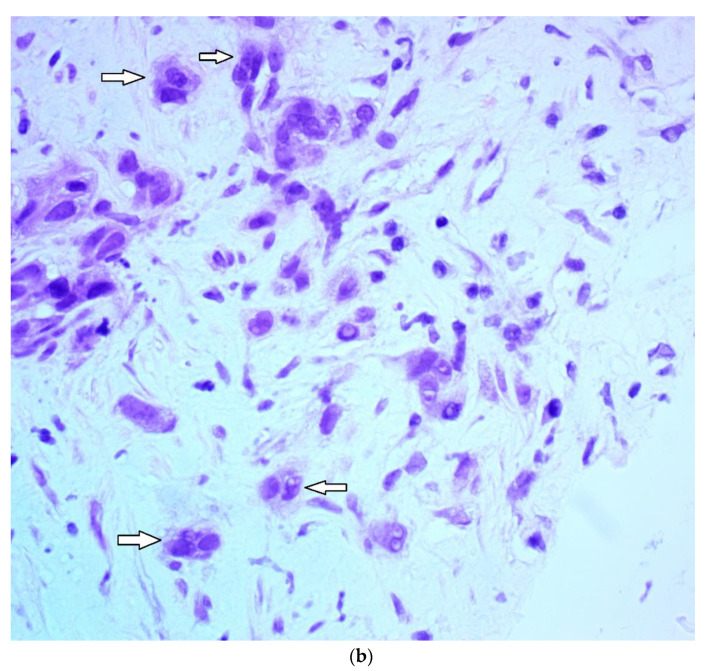
Microphotograph examples of a patient with low-grade budding (**a**) and a patient with high-grade budding (**b**). Arrows pinpoint representative buds.

Tumor budding was examined in eosin and hematoxylin sections of the initial diagnostic biopsy specimens of patients. These specimens were obtained by an interventional radiologist through a core needle biopsy under ultrasound guidance before the initiation of treatment. The presence or absence of budding and the grade (intermediate/high-grade tumor budding: 5 or more buds per 20× power field in a hotspot area of 0.785 mm^2^; low-grade tumor budding: less than 5 buds per 20× power field in a hotspot area of 0.785 mm^2^) were reviewed with the same microscope by two experienced general pathologists and recorded in the study database. We elected to use a 5-bud limit for the categorization of tumor budding grade based on the recommendations of the International Tumor Budding Consensus Conference for colorectal cancer [17]. In case of a discrepancy between the two pathologists, the overall result was decided by consensus. The agreement between pathologists was excellent (kappa statistic 0.89) for the presence or absence of budding and very good (kappa statistic 0.70) for the degree (grade) of budding. A bud was defined as 1 isolated tumor cell or up to 5 cells in clusters detached from the main tumor mass (Figure 1).

Responses to treatment outcomes were recorded for the surgical pathology specimens as follows: a complete response (CR) was defined as no residual tumor in the breast or lymph nodes after neo-adjuvant chemotherapy, a partial response (PR) was defined as minimal or moderate residual disease and evidence of response to therapy, and no response (NR) was defined as extensive residual disease in the breast and/or the lymph nodes without evidence of therapy effect on tumor cells. This practical, three-tier, semiquantitative response evaluation was used based on the ease of evaluation and the fact that different degrees of partial response to therapy forecast an intermediate prognosis between complete response and no response.

Statistical analysis was performed with the Fisher exact test or the *x*^2^ test for comparison of ratios and the *t*-test for comparison of mean differences of continuous variables. Overall survival was evaluated with the Kaplan–Meier method. The Log Rank test was used to compare Kaplan–Meier survival curves. All statistical comparisons were considered significant if *p* < 0.05.

The protocol of the study obtained approval from the Ethics review board of the institution.

## 3. Results

Seventy-five breast cancer patients who received neo-adjuvant chemotherapy were included in the study (Table 1). All patients were women, and the mean age was 58.9 year. Most patients in the series (69.3%) were younger than 65 years old, and most patients (74.7%) were post-menopausal. Three patients had stage I disease, and the rest had stage II or stage III cancer. Most patients had ER-positive cancers (77.3%) and HER2-negative cancers (69.3%). The sub-type distribution was as follows: 54.3% of patients had ER-positive/HER2-negative cancers, 32% of patients had HER2-positive cancers, and 13.3% of patients had triple negative breast cancers. About four out of five patients had ductal carcinomas. Two-thirds of patients were positive for tumor budding. There were no significant differences in age, menopause status, stage, ER, PR or HER2 positivity, or overall sub-type between patients with and without tumor budding (Table 1).

Table 2 shows the presence or absence of tumor budding according to histologic type and immunohistochemical sub-type of breast cancers. As expected, all but one of the lobular cancers were ER-positive, and most (80%) had tumor budding.

The majority of patients in the series (63 patients, 84%) received neo-adjuvant chemotherapy with the FEC-D regimen (3 cycles of 5-FU, epirubicin and cyclophosphamide followed by 3 cycles of docetaxel every 3 w). Other regimens used included AC-paclitaxel (4 cycles of adriamycin with cyclophosphamide followed by 4 cycles of paclitaxel every 2 w) in 4 patients, docetaxel-cyclophosphamide for 4 cycles every 3 w in 2 patients, carboplatin-docetaxel for 6 cycles every 3 w in 3 patients and weekly paclitaxel monotherapy for 12 weeks in 1 patient (Table 3). All HER2-positive patients received concomitant trastuzumab in cycles not containing an anthracycline. Two patients with ER-positive and HER2-negative cancers received hormonal therapy as neo-adjuvant treatment (one received letrozole and the other anastrozole). One of these patients was diagnosed with a colon cancer and did not have her breast cancer excised, and the other had no pathologic response at surgery.

Among the 75 patients, 72 completed neo-adjuvant chemotherapy and underwent surgical resection of their breast cancer. Fifteen of the 72 patients (20.8%) had CR, 20 patients (27.8%) had PR and 37 patients (51.4%) had NR (Table 1). CR rates were 7.5% in ER-positive/HER2-negative patients, 40.9% in HER2-positive patients and 30% in triple negative patients. There were no statistically significant differences in the response to neo-adjuvant therapy according to the presence or absence of tumor budding in pre-operative biopsies (Table 1). Although lobular ER-positive cancers had numerically higher rates of tumor budding than ductal ER-positive carcinomas (78.6% versus 61.9%), the difference was not statistically significant, possibly due to small numbers (Table 2).

Among patients with tumor budding, 36 patients (72%) had intermediate-/high-grade tumor budding (five or more buds per hotspot high-power field) and 14 patients (28%) had low-grade tumor budding. The degree of tumor budding was not associated with response to therapy (Table 4).

**Figure 2:**
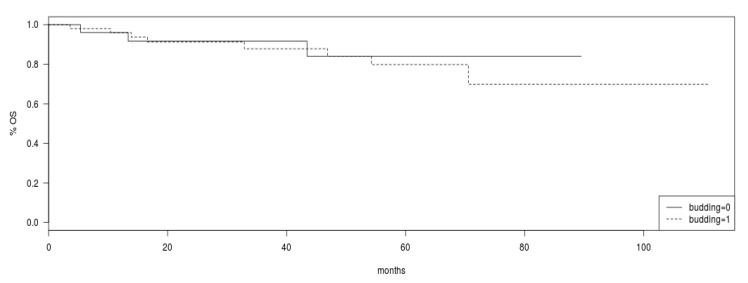
Overall survival (OS) in the group of patients with tumor budding (intermittent line) versus patients without tumor budding (continuous line). Log Rank *p* = 0.8.

Overall survival was not different between patients with tumor budding and patients with absence of tumor budding in pre-operative biopsies (Log Rank test *p* = 0.8, Figure 2).

## 4. Discussion

Tumor budding in cancer is defined as isolated cancer cells or small clusters of cancer cells separated from the main tumor mass, seen in histologic sections [8]. From a pathophysiologic point of view, tumor budding represents an initial stage of the metastatic process. During metastasis, cells that have acquired metastatic potential detach from neighboring cells and start to move away from the main tumor [7,12]. As metastatic potential develops, epithelial cells obtain mesenchymal features through a process called epithelial-to-mesenchymal transition (EMT). At the metastatic site in remote organs, cancer cells regain epithelial characteristics through the reverse process, termed mesenchymal-to-epithelial transition (MET) [18,19]. EMT and MET, collectively called epithelial mesenchymal plasticity, are physiologic processes of normal embryogenesis, organogenesis and wound healing and have been usurped by cancer cells. EMT and MET in cancer are often incomplete, and cells expressing epithelial or mesenchymal markers may be part of a continuous spectrum of states [20,21]. In addition, EMT and MET in cancer have been recognized to empower cancer cells with stem cell properties [22]. The plasticity of stem cells allows motile cells in transit to alternate between the epithelial and mesenchymal state during their metastatic journey from the primary site to the metastatic site [23,24].

As a putative depiction of the initial steps of the metastatic cascade, presence of tumor budding has been found to be of prognostic significance in various cancers, including carcinomas of gastrointestinal origin, lung cancer, head and neck carcinomas, and breast cancer [8,25,26,27]. Colorectal cancers are the type of primary cancers where tumor budding was initially studied and remain the best studied locations [8]. In stage II operated colorectal cancers, patients with tumor budding had worse survival outcomes at 5 y compared with counterparts that had no tumor budding [10]. In rectal cancer, the presence of tumor budding in biopsies before neo-adjuvant chemo-radiation was an adverse predictive factor for response to therapy [11]. In squamous esophageal cancer, the presence of tumor budding in surgical resection specimens after neo-adjuvant chemo-radiation was predictive of worse outcomes [23]. Similarly, in resected stage I lung cancer, patients with high-grade budding had a higher recurrence rate than patients with low-grade budding [25]. In early breast cancer, tumor budding has been reported to predict worse overall survival (OS) in triple negative but not in ER-positive and HER2-negative disease [13].

This study reports on the associations and predictive implications of tumor budding in a series of breast cancer patients that received neo-adjuvant therapy and had a diagnostic core needle biopsy before staring neo-adjuvant treatment. These biopsies yielded sufficient material for budding evaluation in all cases in this series. The feasibility of budding evaluation was consistent with the experiences in colorectal cancer literature, where endoscopic biopsies have been used for rectal cancer evaluation before neo-adjuvant chemoradiation [11]. Two-thirds of breast cancer patients with localized cancers that necessitated neo-adjuvant therapy had some degree of tumor budding in the initial biopsy, most of them (72%) were high-grade budding, defined as five or more buds per high-power field at a hotspot area of the biopsy. Patients with HER2-positive and triple negative cancers had higher pathologic complete response rates at surgery than patients with ER-positive/HER2-negative cancers. However, no correlations of tumor budding in the pre-operative biopsy with response to therapy or with tumor characteristics were evident. These results suggest that tumor budding is not a reliable marker of response to neo-adjuvant therapy in breast cancer patients. In contrast to these results in the neo-adjuvant setting of breast cancer, tumor budding has been predictive of response to therapy in rectal cancer patients receiving neo-adjuvant chemoradiation. This suggests that the implications of similar pathologic phenomena may vary in different cancers [11]. Despite this discrepancy, the prognostic significance of tumor budding has also been suggested in sub-sets of breast cancer [13]. A series of ER-positive, HER2-negative and triple negative localized breast cancers showed that presence of tumor budding was associated with worse OS in triple negative patients, while no prognostic value was discerned in ER-positive and HER2-negative patients [13]. While tumor budding was not prognostic for disease-free survival (DFS) in either group, it was prognostic for worse DFS in the sub-group of ER-positive and HER2-negative patients with an intermediate Oncotype Dx score. Another study in early breast cancer suggested that both tumor budding of less than five cells and of more than five cells not forming glands (the latter termed “poorly differentiated clusters” by the authors of the study) were prognostic for overall and disease-free survival [28]. The authors suggested that poorly differentiated clusters were the preferred biomarker given that they were more easily identifiable [28]. The results of these studies imply that the prognostic value of tumor budding in breast cancer is sub-type and risk group-specific. A meta-analysis of seven studies of tumor budding in breast cancer found that high-grade tumor budding was associated with lymph node positivity and lymphovascular invasion, while high-grade tumor budding had a reduced prevalence in triple negative breast cancers [29]. Studies included in this meta-analysis used different cut-offs to define high-grade budding, including number of buds and fields that were examined for tumor budding grading [29,30]. This heterogeneity of definitions decreased the confidence regarding the observed associations. It also impeded development of tumor budding as a clinical marker in this setting.

The current study is the first to explore tumor budding in the neo-adjuvant setting. It confirmed that tumor budding is a common pathologic occurrence in breast cancer and is associated with all molecular sub-types. There was no association of tumor budding with response to neo-adjuvant treatment in this series. However, due to the comparatively small size of the cohort, associations of tumor budding with response to therapy in specific breast cancer sub-types, such as triple negative cancers as observed in the post-operative setting by other investigators, were not excluded. As there were only 10 triple negative patients in the current series, the lack of association of tumor budding with response to therapy should be interpreted with caution in this group. Other limitations of this study include the retrospective design and the fact that it was performed at a single center, with evaluations performed by two experienced pathologists. As a result, whether findings can be replicated in other centers or by pathologists less experienced in breast pathology remains unknown.

Overall, we have shown the common occurrence of tumor budding as a pathologic phenomenon at diagnosis before any treatment in breast cancer. Tumor budding may be identified in eosin and hematoxylin histologic sections without the need of additional stains or special slide preparation. The value of tumor budding as a prognostic marker in specific sub-sets of breast cancer remains an open question that will require more extensive studies. Whether further characterization of budding cells with immunohistochemistry stains for key proteins of the metastatic process may add value to their study as prognostic factors in breast cancer awaits future evaluations.

## Figures and Tables

**Table 1:** Demographic and disease characteristics of the patients in the series and according to presence of tumor budding.

	Category	Total (%) (*n* = 75)	Tumor Budding Absent (*n* = 25)	Tumor Budding Present (*n* = 50)	*p* Value
**AGE**	Mean	58.9	58.9	59	*p* = 0.9
	≤ 65	52 (69.3%)	19 (76%)	33 (66%)	*p* = 0.43
	> 65	23 (30.7%)	6 (24%)	17 (34%)	
**MENOPAUSE STATUS**	Pre-/perimenopausal	19 (25.3%)	7 (28%)	12 (24%)	*p* = 0.78
	Post-menopausal	56 (74.7%)	18 (72%)	38 (76%)	
**CLINICAL STAGE**	I	3 (4%)	1 (4%)	2 (4%)	*p* = 0.26 (Stage I/II versus stage III)
	II	52 (69.3%)	15 (60%)	37 (74%)	
	III	20 (26.7%)	9 (36%)	11 (22%)	
**ER**	positive	58 (77.3%)	20 (80%)	38 (76%)	*p* = 0.77
	negative	17 (22.7%)	5 (20%)	12 (24%)	
**PR**	positive	47 (62.7%)	16 (64%)	31 (62%)	*p* = 0.99
	negative	28 (37.3%)	9 (36%)	19 (38%)	
**HER2**	positive	23 (30.7%)	7 (28%)	16 (32%)	*p* = 0.79
	negative	52 (69.3%)	18 (72%)	34 (64%)	
**SUB-TYPE**	ER+/ HER2−	41 (54.7%)	15 (60%)	26 (52%)	
	HER2+	24 (32%)	8 (32%)	16 (32%)	*p* = 0.6
	Triple Negative	10 (13.3%)	2 (8%)	8 (16%)	
**HISTOLOGY** **(*n* = 74)**	Ductal	58 (78.4%)	21 (84%)	37 (74%)	*p* = 0.35 (Ductal versus lobular/ mixed)
	Lobular	10 (13.5%)	3 (12%)	7 (14%)	
	Mixed	5 (6.8%)	0	5 (10%)	
	Other	1 (1.3%)	1 (4%)	0	
**RESPONSE** **(*n* = 72)**	CR	15 (20.8%)	4 (16.7%)	11 (22.9%)	*p* = 0.8
	PR	20 (27.8%)	7 (29.2%)	13 (27.1%)	
	NR	37 (51.4%)	13 (54.2%)	24 (50%)	

Percentages in the third column refer to the total number of patients, and in the fourth and fifth columns they refer to the groups without and with budding; CR: complete response, PR: partial response, NR: no response.

**Table 2:** Presence of tumor budding according to histologic type (ductal versus lobular/mixed) and molecular sub-type.

	Ductal			Lobular/Mixed		
Sub-Type	Total (%) (*n* = 58)	Tumor Budding Absent (*n* = 21)	Tumor Budding Present (*n* = 37)	Total (%) (*n* = 15)	Tumor Budding Absent (*n* = 3)	Tumor Budding Present (*n* = 12)
ER+/HER2−	29 (50%)	11 (52.4%)	18 (48.7%)	11 (73.3%)	3 (100%)	8 (66.7%)
HER2+/ER+	13 (22.4%)	5 (23.8%)	8 (21.6%)	3 (20%)	0	3 (25%)
HER2+/ ER−	6 (10.4%)	3 (14.3%)	3 (8.1%)	1 (6.7%)	0	1 (8.3%)
TNBC	10 (17.2%)	2 (9.5%)	8 (21.6%)	0	0	0

TNBC: Triple negative Breast Cancer.

**Table 3:** Tumor budding and response to treatment according to chemotherapy regimen received.

	Category	Total (%) (*n* = 69)	Tumor Budding Absent (*n* = 24)	Tumor Budding Present (*n* = 45)
FEC-D (*n* = 61)				
RESPONSE	CR	12 (19.7%)	3 (14.3%)	9 (22.5%)
	PR	17 (27.9%)	6 (28.6%)	11 (27.5%)
	NR	32 (52.4%)	12 (57.1%)	20 (50%)
AC-Paclitaxel (*n* = 4)				
RESPONSE	CR	1 (25%)	1 (100%)	0
	PR	1 (25%)	0	1 (33.3%)
	NR	2 (50%)	0	2 (66.7%)
Carboplatin-Docetaxel (*n* = 3)				
RESPONSE	CR	0	0	0
	PR	2 (66.7%)	1 (50%)	1 (100%)
	NR	1 (33.3%)	1 (50%)	0
Docetaxel-Cyclophosphamide (*n* = 1)				
RESPONSE	CR	1 (100%)	0	1 (100%)
	PR	0	0	0
	NR	0	0	0

One patient, not included in the table, had tumor budding and obtained a complete pathologic response after receiving paclitaxel monotherapy. Two patients had hormonal therapy. One patient did not have surgery, and the second had tumor budding and had no response to the neo-adjuvant hormonal treatment. Among the two patients that had the docetaxel-cyclophosphamide regimen, one patient had no surgery. Percentages are for each regimen. Patients with HER2+ cancers received trastuzumab with their chemotherapy. CR: complete response, PR: partial response, NR: no response.

**Table 4:** Demographic and disease characteristics of the patients in the series and according to presence of intermediate/high tumor budding (≥5 buds per 20× power field in a hotspot area of 0.785 mm^2^).

	Category	Total (%) (*n* = 75)	Low Tumor Budding (*n* = 39)	Intermediate/High Tumor Budding (*n* = 36)	*x* ^2^
AGE	Mean	58.9	59.1	58.8	*p* = 0.8
	≤65	52 (69.3%)	28 (71.8%)	24 (66.7%)	*p* = 0.8
	>65	23 (30.7%)	11(28.2%)	12 (33.3%)	
MENOPAUSE STATUS	Pre-/perimenopausal	19 (25.3%)	11 (28.2%)	8 (22.2%)	*p* = 0.7
	Post-menopausal	56 (74.7%)	28 (71.8%)	28 (77.8%)	
CLINICAL STAGE	I	3 (4%)	1 (2.6%)	2 (5.6%)	*p* = 0.6
	II	52 (69.3%)	26 (66.7%)	26 (72.2%)	
	III	20 (26.7%)	12 (30.7%)	8 (22.2%)	
ER	positive	58 (77.3%)	29 (74.4%)	29 (80.6%)	*p* = 0.7
	negative	17 (22.7%)	10 (25.6%)	7 (19.4%)	
PR	positive	47 (62.7%)	24 (61.5%)	23 (63.9%)	*p* = 0.8
	negative	28 (37.3%)	15 (38.5%)	13 (36.1%)	
HER2	positive	23 (30.7%)	12 (30.7%)	11 (30.6%)	*p* = 0.9
	negative	52 (69.3%)	27 (69.3%)	25 (69.4%)	
SUB-TYPE	ER+/ HER2−	41 (54.7%)	22 (56.4%)	19 (52.7%)	
	HER2+	24 (32%)	13 (33.3%)	11 (30.6%)	*p* = 0.7
	Triple Negative	10 (13.3%)	4 (10.3%)	6 (16.7%)	
HISTOLOGY (*n* = 74)	Ductal	58 (78.4%)	32 (82%)	26 (74.3%)	
	Lobular	10 (13.5%)	4 (10.3%)	6 (17.1%)	*p* = 0.7
	Mixed	5 (6.8%)	2 (5.1%)	3 (8.6%)	
	Other	1 (1.3%)	1 (2.6%)	0	
RESPONSE (*n* = 72)	CR	15 (20.8%)	8 (21%)	7 (20.6%)	*p* = 0.69
	PR	20 (27.8%)	9 (23.7%)	11 (32.3%)	
	NR	37 (51.4%)	21 (55.3%)	16 (47.1%)	

## Data Availability

There are no data available beyond the results presented in the paper.

## References

[1] Siegel R.L., Miller K.D., Jemal A. (2020). Cancer statistics, 2020. CA Cancer J. Clin..

[2] DeSantis C.E., Ma J., Gaudet M.M., Newman L.A., Miller K.D., Goding Sauer A., Jemal A., Siegel R.L. (2019). Breast cancer statistics, 2019. CA Cancer J. Clin..

[3] Cady B., Fulton J.P. (2020). 57% decline in Rhode Island invasive breast cancer mortality between 1987 and 2017: Mammography predominates in preventing mortality. Breast Cancer Res. Treat..

[4] Battisti N.M.L., Tong D., Ring A., Smith I. (2019). Long-term outcome with targeted therapy in advanced/metastatic HER2-positive breast cancer: The Royal Marsden experience. Breast Cancer Res. Treat..

[5] Houssami N., Macaskill P., von Minckwitz G., Marinovich M.L., Mamounas E. (2012). Meta-analysis of the association of breast cancer subtype and pathologic complete response to neoadjuvant chemotherapy. Eur. J. Cancer.

[6] Rouzier R., Perou C.M., Symmans W.F., Ibrahim N., Cristofanilli M., Anderson K., Hess K.R., Stec J., Ayers M., Wagner P. (2005). Breast cancer molecular subtypes respond differently to preoperative chemotherapy. Clin. Cancer Res..

[7] Voutsadakis I.A. (2018). Prognostic role of tumor budding in breast cancer. World J. Exp. Med..

[8] Dawson H., Lugli A. (2015). Molecular and pathogenetic aspects of tumor budding in colorectal cancer. Front. Oncol..

[9] Canguçu A.L., Valério E., Peixoto R.B.P., Felismino T.C., de Mello C.A.L., Neotti T., Calsavara V.F., de Macedo M.P., Júnior S.A., Riechelmann R. (2020). The prognostic influence of tumour budding in Western patients with stage II colorectal cancer. Ecancermedicalscience.

[10] Petrelli F., Pezzica E., Cabiddu M., Coinu A., Borgonovo K., Ghilardi M., Lonati V., Corti D., Barni S. (2015). Tumour budding and survival in stage II colorectal cancer: A systematic review and pooled analysis. J. Gastrointest. Cancer.

[11] Rogers A.C., Gibbons D., Hanly A.M., Hyland J.M., O’Connell P.R., Winter D.C., Sheahan K. (2014). Prognostic significance of tumor budding in rectal cancer biopsies before neoadjuvant therapy. Mod. Pathol..

[12] Grigore A.D., Jolly M.K., Jia D., Farach-Carson M.C., Levine H. (2016). Tumor budding: The name is EMT. Partial, EMT.. J. Clin. Med..

[13] Li X., Wei B., Sonmez C., Li Z., Peng L. (2017). High tumor budding count is associated with adverse clinicopathologic features and poor prognosis in breast carcinoma. Hum. Pathol..

[14] Liang F., Cao W., Wang Y., Li L., Zhang G., Wang Z. (2013). The prognostic value of tumor budding in invasive breast cancer. Pathol. Res. Pract..

[15] Gujam F.J.A., McMillan D.C., Mohammed Z.M.A., Edwards J., Going J.J. (2015). The relationship between tumour budding, the tumour microenvironment and survival in patients with invasive ductal breast cancer. Br. J. Cancer.

[16] Salhia B., Trippel M., Pfaltz K., Cihoric N., Grogg A., Lädrach C., Zlobec I., Tapia C. (2015). High tumor budding stratifies breast cancer with metastatic properties. Breast Cancer Res. Treat..

[17] Lugli A., Kirsch R., Ajioka Y., Bosman F., Cathomas G., Dawson H., El Zimaity H., Fléjou J.F., Hansen T.P., Hartmann A. (2017). Recommendations for reporting tumor budding in colorectal cancer based on the International Tumor Budding Consensus Conference (ITBCC) 2016. Mod Pathol..

[18] Kalluri R., Weinberg R.A. (2009). The basics of epithelial-mesenchymal transition. J. Clin. Investig..

[19] Voutsadakis I.A. (2016). Epithelial-Mesenchymal Transition (EMT) and regulation of EMT factors by steroid nuclear receptors in breast cancer: A review and in silico investigation. J. Clin. Med..

[20] Lambert A.W., Pattabiraman D.R., Weinberg R.A. (2017). Emerging biologic principles of metastasis. Cell.

[21] Voutsadakis I.A. (2012). The Ubiquitin–Proteasome System and signal transduction pathways regulating Epithelial Mesenchymal transition of cancer. J. Biomed. Sci..

[22] Voutsadakis I.A. (2015). The network of pluripotency, epithelial-mesenchymal transition, and prognosis of breast cancer. Breast Cancer.

[23] Mani S.A., Guo W., Liao M.J., Eaton E.N., Ayyanan A., Zhou A.Y., Brooks M., Reinhard F., Zhang C.C., Shipitsin M. (2008). The epithelial-mesenchymal transition generates cells with properties of stem cells. Cell.

[24] Morel A.P., Lièvre M., Thomas C., Hinkal G., Ansieau S., Puisieux A. (2008). Generation of breast cancer stem cells through epithelial-mesenchymal transition. PLoS ONE.

[25] Kadota K., Yeh Y.C., Villena-Vargas J., Cherkassky L., Drill E.N., Sima C.S., Jones D.R., Travis W.D., Adusumilli P.S. (2015). Tumor budding correlates with the protumor immune microenvironment and is an independent prognostic factor for recurrence of stage I lung adenocarcinoma. Chest.

[26] Shimizu S., Miyazaki A., Sonoda T., Koike K., Ogi K., Kobayashi J., Kaneko T., Igarashi T., Ueda M., Dehari H. (2018). Tumor budding is an independent prognostic marker in early stage oral squamous cell carcinoma: With special reference to the mode of invasion and worst pattern of invasion. PLoS ONE.

[27] Lugli A., Zlobec I., Berger M.D., Kirsch R., Nagtegaal I.D. (2020). Tumour budding in solid cancers. Nat. Rev. Clin. Oncol..

[28] Sun Y., Liang F., Cao W., Wang K., He J., Wang H., Wang Y. (2014). Prognostic value of poorly differentiated clusters in invasive breast cancer. World J. Surg. Oncol..

[29] Lloyd A.J., Ryan É.J., Boland M.R., Elwahab S.A., Malone C., Sweeney K.J., Barry K.M., McLaughlin R., Kerin M.J., Lowery A.J. (2020). The histopathological and molecular features of breast carcinoma with tumour budding-a systematic review and meta-analysis. Breast Cancer Res Treat..

[30] Agarwal R., Khurana N., Singh T., Agarwal P.N. (2019). Tumor budding in infiltrating breast carcinoma: Correlation with known clinicopathological parameters and hormone receptor status. Indian J. Pathol. Microbiol..

